# Nuclear Coregulatory Complexes in Tregs as Targets to Promote Anticancer Immune Responses

**DOI:** 10.3389/fimmu.2022.909816

**Published:** 2022-06-20

**Authors:** Lanette M. Christensen, Wayne W. Hancock

**Affiliations:** ^1^Department of Pathology and Laboratory Medicine, Children’s Hospital of Philadelphia, Philadelphia, PA, United States; ^2^Department of Pathology and Laboratory Medicine, University of Pennsylvania, Philadelphia, PA, United States

**Keywords:** histone/protein deacetylases, inhibitors, tumor immunity, T-regulatory cells, SIN3A

## Abstract

T-regulatory (Treg) cells display considerable heterogeneity in their responses to various cancers. The functional differences among this cell type are heavily influenced by multiprotein nuclear complexes that control their gene expression. Many such complexes act mechanistically by altering epigenetic profiles of genes important to Treg function, including the forkhead P3 (Foxp3) transcription factor. Complexes that form with certain members of the histone/protein deacetylase (HDAC) class of enzymes, like HDACs 1, 2, and 3, along with histone methyltransferase complexes, are important in the induction and stabilization of Foxp3 and Treg identity. The functional behavior of both circulating and intratumoral Tregs greatly impacts the antitumor immune response and can be predictive of patient outcome. Thus, targeting these regulatory complexes within Tregs may have therapeutic potential, especially in personalized immunotherapies.

## Introduction

The immune system requires the cooperation of complex cellular and molecular signaling pathways to perform its functions and maintain homeostasis. Various immune cell types are specialized in the performance of these diverse functions, including T-regulatory (Treg) cells, which limit immune responses and help maintain self-tolerance (1–3). The function of Tregs depends on the regulation and manipulation of their gene expression. With few exceptions, the expression of forkhead P3 (Foxp3), CD25, CTLA4, HELIOS, and GITR defines the Treg lineage identity (4), although various subsets of Tregs display additional characteristics (5). The expression of Foxp3, the master regulator component of Treg transcription (6, 7), is controlled in multiple ways, including epigenetic mechanisms. Histone methylation, acetylation, ubiquitination, and DNA methylation all contribute to the epigenetic regulation of Foxp3+ Tregs, both by direct regulation of Foxp3 expression and by regulation of the total Foxp3+ Treg transcriptional identity (8). A consistent epigenetic feature of Tregs with stable Foxp3 expression is hypomethylation at the conserved non-coding sequence 2 (CNS2 or Treg-specific demethylation region, aka TSDR) site (9). Foxp3+ Tregs that lack a stable epigenetic profile can lose Foxp3 and gain proinflammatory IL-17 expression, and IL-17-producing Tregs contribute to the pathology of multiple inflammatory diseases (10, 11). Histone modification, contrary to DNA modification, tightly controls more transient regulation of gene expression.

**Figure 1:**
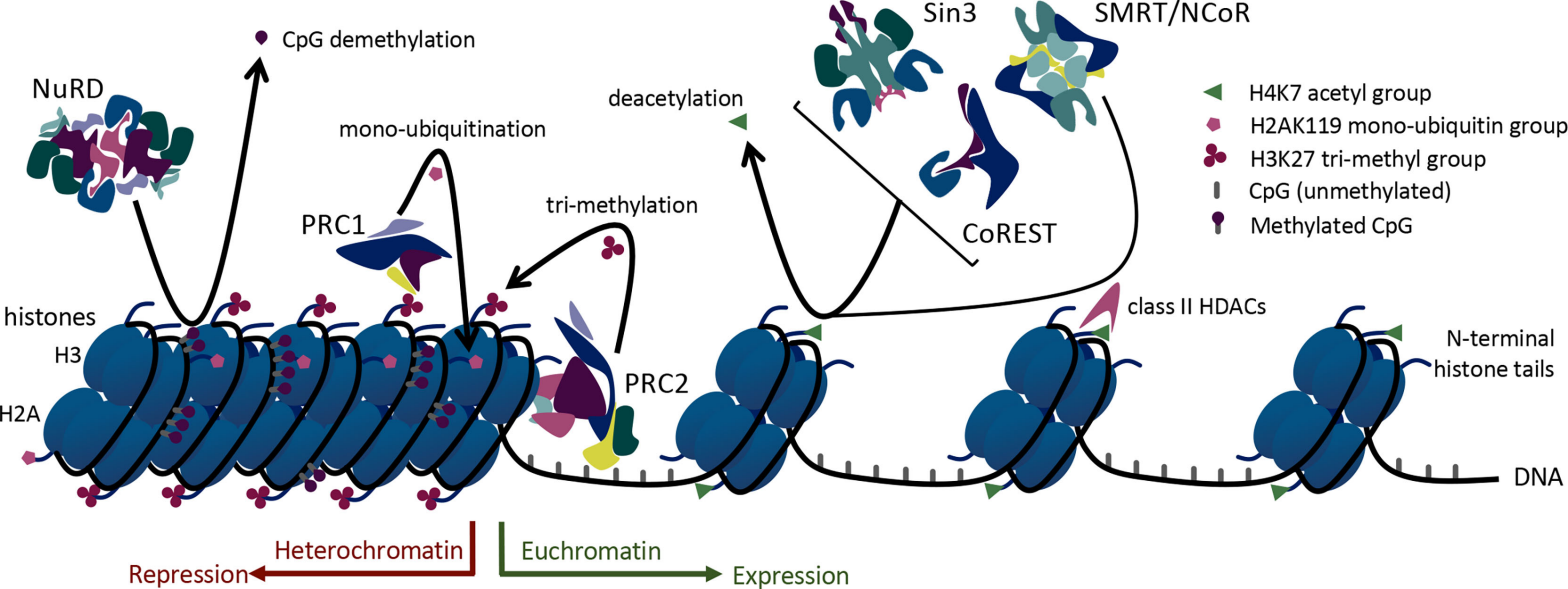
Mechanisms of epigenetic regulation utilized by large multiprotein complexes in Treg cells.

Foxp3+ Tregs rely upon histone modification *via* acetylation, methylation, and ubiquitination to regulate and shift their acute transcriptional functions. Histone acetylation occurs on lysine residues of histones and leads to less condensed chromatin (euchromatin), thus allowing access of transcription factors to the DNA. Enzymes with opposing actions, histone/protein acetyltransferases (HATs) and histone/protein deacetylases (HDACs), are responsible for the addition or removal of acetyl groups on histones (12). Higher HAT activity and lower HDAC activity support transcriptional gene expression. Consistent with that, chromatin surrounding actively transcribed Foxp3 genes is hyperacetylated on histones H3 and H4 (13) (**Figure 1**). Histone acetylation has an important role in Foxp3 regulation mediated by three HATs and many HDACs (13, 14). These enzymes can also catalyze post-translational modifications of Foxp3. Thus, 3 HATs (CBP, p300 and TIP60, and p300/CBP) play critical roles in Foxp3 acetylation and promote Treg function (15–17), whereas at least 12 HDACs, (HDACs 1, 2, 3, 5, 6, 7, 8, 9, 10, and 11 and SIRT1 and SIRT3) can catalyze Foxp3 deacetylation with varying effects on Treg function (18–22) (**Table 1**).

**Table 1:** Effects of HDAC targeting in Treg cells.

HDAC	HDAC class	Effect of HDAC gene deletion or knockdown on Treg function	Specific pharmacologic inhibitor(s) available	Reference
HDAC1	I	Increased	No	(23)
HDAC2	I	Decreased	No	(23)
HDAC3	I	Decreased	Yes	(24)
HDAC5	IIa	Decreased	Yes	(25)
HDAC6	IIb	Increased	Yes	(19)
HDAC7	IIa	Increased	No	(26)
HDAC8	I	Decreased	Yes	(27)
HDAC9	IIa	Increased	No	(28)
HDAC10	IIb	Increased	Yes	(22)
HDAC11	IV	Increased	Yes	(29)
**SIRT**				
SIRT1	III	Increased	Yes	(20)
SIRT2	III	No effect	No	(30)
SIRT3	III	Decreased	Yes	(31)

HDAC, histone deacetylase; SIRT, sirtuin.

**Figure 2:**
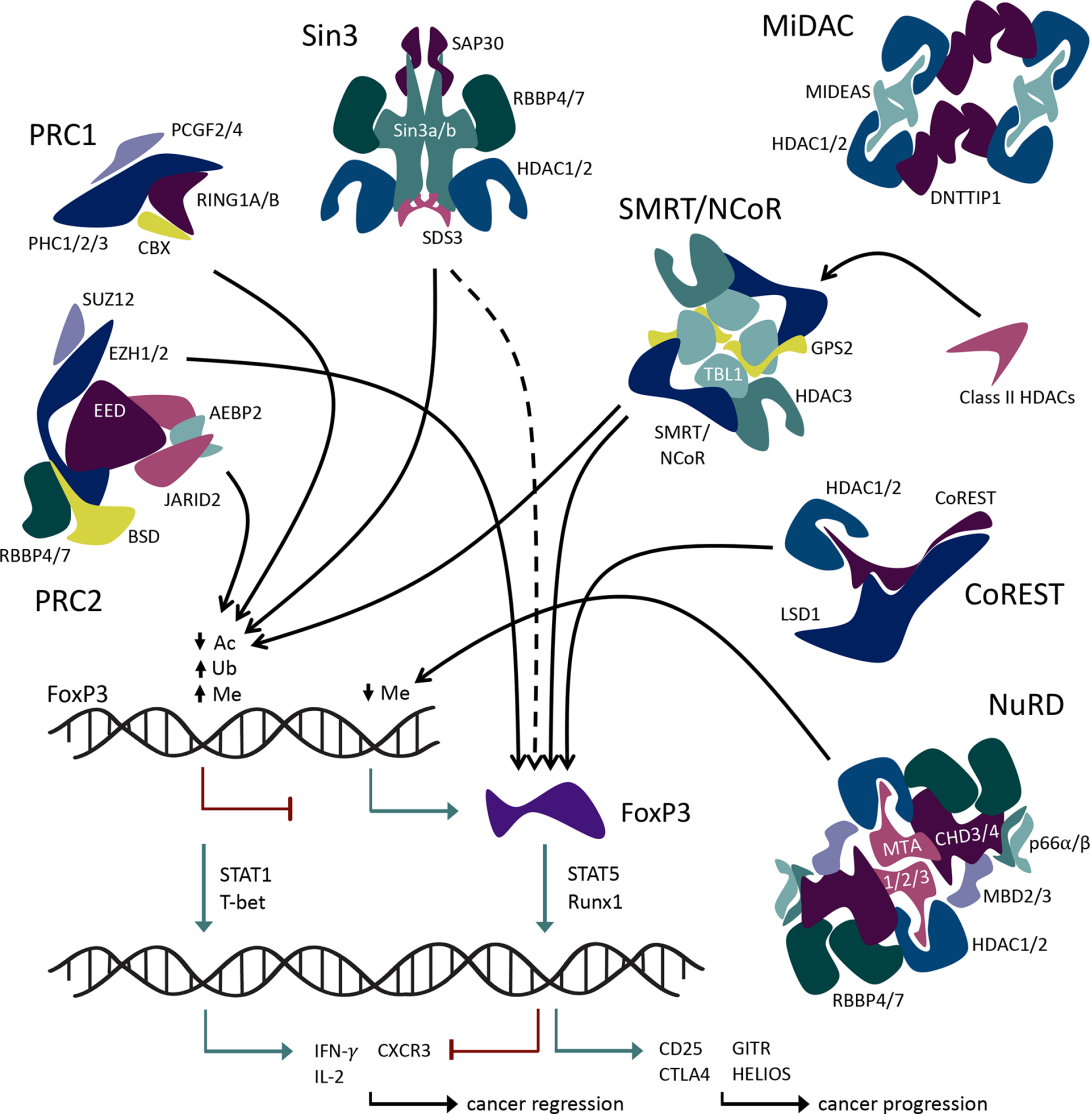
Interactions of large multiprotein coregulatory complexes with Foxp3.

The 18 known HDACs are organized into 5 classes. HDACs involved in Foxp3 regulation come from all 5 HDAC classes: class I (HDACs 1, 2, 3, and 8), class IIa (HDACs 5, 7, and 9), class IIb (HDACs 6 and 10), class IV (HDAC11), and class III (SIRT1, 2 and 3). Class I, II, and IV HDACs have Zn^2+^-dependent catalytic activity (32), whereas class III sirtuins are NAD^+^-dependent and/or ADP ribosylase enzymes (33). Some HDACs, especially class I members, form large protein complexes that are recruited by methyl-binding domain proteins to selectively bind to methylated cytosines, which are frequently observed in the promoter regions of silenced genes (**Figure 2**). Such regulatory complexes include the switch independent 3 (Sin3), nucleosome remodeling and deacetylase (NuRD), mitotic deacetylase (MiDAC), a corepressor of REST (CoREST), and silencing mediator for retinoid or thyroid-hormone receptors/nuclear receptor corepressor (SMRT/NCoR). The Sin3, NuRD, MiDAC, and CoREST complexes include HDAC1 and HDAC2 as the core enzymatically active component(s), while SMRT/NCoR utilizes HDAC3.

In addition to these regulatory complexes that modify histone acetylation, other epigenetic regulatory complexes influence histone methylation and ubiquitination. For example, members of the large multiprotein polycomb repressive complexes (PRCs) help control the methylation and ubiquitination of Foxp3. PRCs 1 and 2 are involved in epigenetic regulation of transcription *via* ubiquitin ligase and methyltransferase activities, respectively. Both histone methylation and ubiquitination *via* PRCs lead to chromatin condensation and transcriptional repression (34) (**Figure 1**). In addition, non-enzymatic chromatin remodeling functions of PRC1 can activate or repress genes, at least in *Drosophila* (35). Like the HDAC-associated multiprotein regulatory complexes, PRC2/1 influences the transcriptional availability of genes important to Tregs including Foxp3 itself, thus influencing the development and stability of Treg cells (**Figure 2**).

These large regulatory complexes contribute to a wide array of cellular functions and processes such as cell cycle, DNA repair and replication, embryo development, stem cell lineage determination, and cell lineage maintenance in diverse tissues and cell types. The activity of most of these epigenetic regulatory complexes typically results in transcriptional repression, and some complexes are exclusively considered corepressors. However, some are coregulators, as they influence both transcriptional activation and repression. Epigenetic coregulatory complexes contribute to genetic regulation within Foxp3+ Tregs both by direct epigenetic modification of Foxp3 promotor TSDR/CNS2 and by participating, in coordination with Foxp3 protein, in establishing and maintaining the Treg transcriptional program (8).

A primary function of Treg cells is to suppress the activity of effector immune cells. In the context of cancer pathology, increased activity of Tregs leads to increased tumor growth and poor patient outcomes (36). Thus, inhibition of Treg function is of interest with respect to developing new anticancer therapies, though options to do so using isoform-selective HDAC inhibitors are basically limited to HDAC3 and HDAC8. By contrast, HDACs 1 and 2, which share 83% identity, are key enzymatic components of several coregulator complexes, often in conjunction with additional enzymes. Within Tregs, histone-modifying, multiprotein regulatory complexes that contribute to controlling the Treg transcriptional program offer promising targets to inhibit suppressive function and will be reviewed.

## CoREST

The CoREST complex is a chromatin-modifying transcriptional coregulator that contains two enzymes with different catalytic activities. In addition to the deacetylase activity of HDAC 1 or 2, the CoREST complex includes lysine-specific demethylase (LSD1). LSD1 targets mono- and di-methylated H3K4 and H3K9. Both catalytic functions can be executed by the CoREST complex, although not necessarily simultaneously (37, 38). HDAC1 or its close paralog HDAC2, together with LSD1 and the scaffolding protein, CoREST1/2/3 (aka RCOR1/2/3), form the overall CoREST complex (39, 40). Homology indicates that the ELM2-SANT domain of CoREST1/2/3 recruits HDAC1/2 and the SANT2 domains of CoREST1/2/3 are proposed to directly interact with DNA (40). The CoREST complex regulates gene expression in neuronal cells and dictates the fate of neuronal stem cells (41). It also has a well-established role in the epigenetic regulation of the hematopoietic system during embryonic development (42).

Transcriptional regulation within Tregs relies on CoREST-mediated repression of proinflammatory genes. Tregs that lack CoREST (Rcor1) undergo functional reprogramming through upregulation of proinflammatory transcription factors, cytokines, and chemokine receptors, including STAT1, T-bet, IL-2, IFN-γ, and CXCR3, mediated by increased H3K9-acetylation and H3K4-dimethylation through reduced recruitment of histone-modifying enzymes to the promotors of proinflammatory genes (43). Diminished suppressive and anti-inflammatory functions accompany the enhanced proinflammatory characteristics of Tregs lacking CoREST (44). Deletion of HDAC1 in Tregs leads to impaired function, while deletion of HDAC2 enhances function (27). HDAC inhibitors that bind both HDAC1 and HDAC2 with differing dissociation constants show relative HDAC2 selectivity (45, 46) and promote Treg functions *in vitro*, but their use has not been explored *in vivo* (23). In contrast, when the CoREST complex is inhibited *via* dual pharmacologic targeting of HDAC and LSD1 enzymes, Treg function is significantly curtailed *in vitro* and *in vivo*. Indeed, the use of corin (a dual-activity CoREST inhibitor) impaired Foxp3+ Treg function and promoted antitumor immunity in murine models (43, 44). A newly described non-canonical role of CoREST associating with RNA Polymerase II during transcription and deacetylating its carboxy-terminal domain at lysine 7 to inhibit productive elongation may also contribute to CoREST activity (47). Inhibition of the CoREST complex by various methods is a promising area of interest for cancer immunotherapy.

## NuRD

The NuRD complex is an ATP-dependent chromatin remodeling large multiprotein complex involved in the regulation of gene transcription, genome stability, cell cycle progression, and DNA damage replication and repair response (48, 49). The core of the NuRD complex is composed of HDAC1/2, metastasis-associated proteins (MTA1/2/3), methyl-CpG binding domain protein 2/3 (MBD2/3), and retinoblastoma-binding proteins (RBBP4/7) in an elongated zigzag conformation (50). Two HDAC1/2 components form a dimer mediated by the ELM2-SANT domains of MTA1, and four RBBP4/7 proteins bind the C terminuses of the dimerized MTA1 proteins; two RBBP4/7 proteins bind one MTA1 protein (51). MBD2 and MTA1 subunits mediate NuRD complex binding to methylated DNA (52, 53). Other more transient components of the complex include p66alph/beta, deleted in oral cancer 1 (DOC1), and CHD3/4 (54–56). CHD3/4 subunits of the NuRD complex are responsible for its chromatin remodeling function *via* ATP-dependent helicase activity, while the HDAC1/2 subunits are responsible for its deacetylase activity (50). LSD1, a core component of the CoREST complex, has also been shown to associate with NuRD in some cell types (51), adding to the versatility and enzymatic potential of this regulatory complex.

The Mi-2/NuRD complex is an abundant deacetylase complex with a broad cellular and tissue distribution and is unique in that it couples histone deacetylation and chromatin-remodeling ATPase activities in the same complex (57). NuRD can perform its many functions using both catalytic and non-catalytic mechanisms. NuRD plus Bcl6 regulates the transcriptional program of both T follicular effector cells and follicular Tregs (54). In another study, the NuRD complex has displayed an unexpected function in its regulation of Foxp3 expression. The MBD2 component of NuRD, which typically mediates DNA-NuRD interaction leading to gene repression, instead promotes the demethylation of Foxp3 TSDR/CNS2 site by recruiting Tet enzymes and enhances Treg function (55). This is currently a mechanism utilized by MBD2/NuRD uniquely within Treg cells and provides a very useful therapeutic target.

## Sin3

The switch-independent proteins, Sin3a and Sin3b, interact with HDACs 1 and/or 2, suppressors of defective silencing 3 (SDS3), sin3a-associated protein p30 (SAP30), FAM60, and RBBP4/7 to form the Sin3 coregulatory complex (58–60). The cofactors structure around Sin3a/b scaffolding proteins (61), which then form dimers through interaction between extended coiled-coil regions of the SDS3 proteins (62). While Sin3a and Sin3b are similar proteins, they can produce varying functions as part of the Sin3 complex (63). HDAC1/2 provides the sole enzymatic functionality of the Sin3 complex and is required for full deacetylase functionality, although its functions expand beyond acetyltransferase activities (64).

Originally described as a transcriptional corepressor (65, 66), the Sin3 complex is now appreciated to also function as a transcriptional activator (67, 68) and is therefore now considered a transcriptional coregulator. Sin3a was first identified in yeast and is highly conserved throughout mammalian species (69). Functions of the Sin3a complex are required for mammalian embryogenesis (70), T-cell lineage development (70), and transcriptional responses to hypoxia (71). In the case of Treg cells, the Sin3 complex interacts with and silences the transcriptional expression of Foxp3 and decreases Treg-suppressive function (72). In addition to silencing Foxp3, emerging research suggests Sin3a influences widespread transcriptional regulation within Tregs, with both inhibitory and enhancing actions (73, 74). The use of Sin3-specific peptide inhibitors in addition to avermectin is effective in impairing tumor growth in models of triple-negative breast cancer (75, 76), demonstrating that the Sin3 complex may be a beneficial target for therapeutic development.

## MiDAC

Mitotic deacetylase complex (MiDAC) is a chromatin remodeling corepressor complex recently identified in affinity chromatography studies of extracts of dividing cells exposed to HDAC inhibitors (77). The MiDAC complex is composed of HDAC1/2, deoxynucleotidyltransferase terminal-interacting protein 1 (DNTTIP1), and mitotic deacetylase-associated SANT domain (MIDEAS) protein and associates with cyclin A2 (CCNA2) and cyclin-dependent kinase (CDK2) (77–79). There are four copies of each protein component of MiDAC within a single complex, where they form a dimer of dimers completing a tetrameric structure. Dimerization of this complex is mediated by the ELM2-SANT domains of MIDEAS along with the N-terminal regions of DNTTIP1 (40). The C-terminal of DNTTIP1 has been suggested to interact directly with DNA and nucleosomes due to its structural relation to SKI/SNO/HDAC (80). MiDAC can modulate gene expression by negatively regulating the repressive histone mark H4K20ac or the active histone mark H3K27ac, respectively, leading to roles as an activator or repressor of different gene sets (81). MiDAC is required for late embryogenesis since deletion of MIDEAS or DNTTIP1 impairs cardiac development and hematopoiesis (82). However, no role for MiDAC in the regulation of immune functions has yet been reported.

## SMRT/NCoR

SMRT and NCoR are homologous non-redundant corepressor proteins that interact with transcriptional repressors and hormone receptors (unbound to ligand). SMRT and NCoR together with transducin beta-like protein 1 (TBL1), G protein pathway suppressor 2 (GPS2), and HDAC3 form the SMRT/NCoR complex (83). The N-termini of TBL1 proteins bind as a tetramer that interacts with the remaining complex proteins SMRT/NCoR, HDAC3, and GPS2 (40). HDAC3 deacetylase activity requires binding to SMRT/NCoR (84). The complex also interacts with class IIa HDACs 4, 5, 7, and 9 (85). Class IIa HDACs have relatively low catalytic activity and act primarily *via* protein–protein interactions and/or participation in large multiprotein complexes such as SMRT/NCoR (86–88). Indeed, the primary role of class IIa HDACs may be to function as acetyl-lysine binding proteins that recruit HDAC3/SMRT/NCoR (89). The SMRT/NCoR repressor complex has significant roles in cardiac and neuronal development and the maintenance of metabolic and immune homeostasis (90). HDAC3 deletion in Tregs derepresses Treg production of IL-2 and is associated with the rapid development of lethal autoimmunity (24). The HDAC3/SMRT/NCoR complex activity is essential for the development and suppressive functions of Foxp3+ Tregs *via* direct Foxp3-HDAC3 interaction (24). Hence, targeting this complex, its interaction with Foxp3, and/or its interaction with class II HDACs is a promising therapeutic strategy for disrupting Treg function for anticancer immunity.

## PRC1/2

PRC1 and PRC2 are epigenetic regulators originally identified in *Drosophila* as important to cell lineage determination and maintenance through transcriptional repression (35). Recent studies have shown PRC1 functioning to anchor activating and enhancing loops (91), broadening understanding of the functions of these complexes. PRC2 binds unmethylated CpG islands of repressed genes, resulting in trimethylation of surrounding histones on H3K27 residue. Canonical PRC1 then recognizes the trimethylated H3K27 (H3K27me3) residues and further represses the gene by monoubiquitination of H3AK119 or by promoting non-enzymatic chromatin condensation (92).

Components of the canonical PRC1 complex include obligate heterodimer ubiquitin ligase ring finger protein 1 (RING1A/B) and nucleosome binding subunit chromo box (CBX), together with polyhomeotic homolog (PHC1, PHC2, and PHC3) and polycomb group ring finger protein (PCGF2, 3, and 4) subunits (40). PRC1 represses gene transcription through enzymatic histone ubiquitination and non-enzymatic chromatin re-structuring and condensation mechanisms (34). RING1A/B catalyzes the ubiquitination of histones H2A on lysine 119 (H2AK119). Core components of PRC2 include enhancer of zeste homolog 1 (EZH1) or EZH2, embryotic ectoderm development (EED), and suppressor of zeste 12 (SUZ12) together with the more transiently associated subunits of AE binding protein 2 (AEBP2), Jumonji and AT-rich interaction domain containing 2 (JARID2), and RBAP46 (aka RBBP7) or RBAP48 (aka RBBP4). Active PRC2 represses gene transcription *via* histone di- or tri-methylation of H3K27. The enzymatically active component of the PRC2 complex consists of the histone methyltransferases, EZH1 or EZH2, which facilitate the addition of methyl groups to histones (92). While PRC1 and PRC2 are often expressed coordinately, they can also function independently in certain cell types.

The Treg master transcription factor, Foxp3, is subject to epigenetic repression by PRC1 and 2 in the classical PRC2/1 repression and maintenance model. PRC-associated elements recruit PRC2 to unmethylated CpG regions of the Foxp3 promoter, resulting in histone methylation of the region and therefore transcriptional repression of Foxp3 (93). PRC1 complex containing the heterodimeric RING1A/B and PCGF4 homolog (BMI1) recognizes PRC2-mediated H3K27me3 of the Foxp3 promoter(s) and functions to maintain its inactivated state *via* ubiquitination of H3AK119 (94). The EZH2 methyltransferase helps maintain Treg identity and function following activation by being recruited to the Foxp3 protein and leading to repression of genes within the Foxp3 transcriptional program (93–96).

PRC subunits tend to be upregulated in cancers such as melanomas, lymphomas, and prostate and breast cancers (41). Mutations, both gain-of-function and loss-of-function, in PRC2 components especially EZH2 can lead to various cancer manifestations. EZH2 dysregulation specifically has been associated with particularly aggressive cancers and malignancies (92). PRC2 complex activity has exhibited oncogenic and tumor-suppressive functions (35, 92). Interestingly, perturbations in PRC2 and/or H3K27me3, which occur in various hematopoietic malignancies, also render the cancerous cells susceptible to PRC2/1 complex inhibition (97). PRC2 could potentially make a very effective target for Treg inhibition because of its role in maintaining the Foxp3-lead transcriptional profile during Treg activation. For the same reason, it would most likely provide a useful therapeutic used in combination with a different functional target.

## Discussion

While Treg cells play an essential role in maintaining a homeostatic balance within the immune system, their suppressive activity impedes the effector immune response to tumors. When Treg function is abrogated, the effector immune response is unrestrained, and anticancer immunity increases. For these reasons, Tregs provide a valuable target for immunotherapies, especially as part of combination therapy. The 5 large epigenetic regulatory complexes discussed in this review provide targets for inhibition within Tregs, given their involvement in the maintenance and stability of Foxp3+ Treg function. The complexes function both by epigenetic modification of the TSDR/CNS2 promotor of Foxp3 and by direct interaction with the Foxp3 protein. Despite only limited evidence about the contributions of these multiprotein complexes in Treg biology, the available data suggest the potential to exploit one or more of these complexes to promote antitumor immunity.

HDAC family members are often overexpressed in human cancers (98), histone H4 is commonly deacetylated in human primary malignancies (99), and low acetylation of histone H3 is a predictor of poor outcomes in pancreatic, breast, gastric, ovarian, prostate, and lung cancers (100, 101). Because of this and their roles in transcriptional regulation, HDAC inhibitors have been tested in various cancer models and clinical trials (**Table 2**), though with widely varying results (101). HDAC inhibitors are effective against the progression of various cancers, especially hematological malignancies such as cutaneous T-cell lymphoma (99). HDAC inhibitors have varying effects on Foxp3 expression and Treg-suppressive functions (**Table 1**) (27, 102). An optimal strategy would be to specifically target individual HDACs within Tregs for anticancer therapeutics. In particular, HDACs 1 and 2 are interchangeably present within NuRD, CoREST, MiDAC, and Sin3 complexes, and when knocked out of Tregs, these two HDACs have opposing effects on Treg function. The lack of HDAC1 decreases Treg-suppressive functions, while HDAC2 increases Treg-suppressive functions (**Table 1**). Thus, a specific inhibitor of HDAC2 would be a promising pharmacologic tool for Treg repression in cancer therapy. Unfortunately, such compounds are yet to be available and have been difficult to generate (40). Efforts to overcome this obstacle have included targeting multiple members of a given complex or blocking interactions between complex members to disrupt their formation. An example of the former is the dual-inhibitor corin, which targets both the HDAC and LSD1 components of the CoREST complex (44). When used to treat mice with TC1 lung tumors, corin decreased Foxp3+ Treg function and promoted antitumor immunity (43, 44). This method of dual-target inhibition could be effective on the NuRD complex by targeting the active domains of the HDAC and MBD2 components. The MBD2 component of NuRD is of particular interest in that the mechanism of activating genetic expression by MBD2/NuRD is unique within Tregs (55).

**Table 2:** Epigenetic regulatory complex components targeted for anticancer therapeutics.

Complex	Component	Activity	Therapeutic	Condition
**FDA approved^1^**				
**HDACs**	**HDACs 1, 2, and 3**	HDAC1, 2, 3, and 10 inhibitor	Chidamide	Peripheral T-cell lymphoma
		Class I, II, and IV HDACi	Belinostat	Peripheral T-cell lymphoma
			SAHA (vorinostat)	Cutaneous T-cell lymphoma
			Romidepsin	Cutaneous and peripheral T-cell lymphoma
		Pan-HDACi	Panobinostat	Multiple myeloma
**PRC2**	**EZH1/2**	EZH2 inhibitor	Tazemetostat	Rare sarcoma and follicular lymphoma
**Current clinical trials^2^**				
**CoREST**	**LSD1**	LSD1 inhibitor	SP-2577	Ewing sarcoma, myxoid liposarcoma, and desmoplastic small round cell tumor
			combination therapies	Ewing sarcoma, myxoid liposarcoma, and myelomonocytic leukemia
			IMG-7289	Thrombocythemia and myelofibrosis
			CC-90011	Leukemia
			combination therapies	Prostatic neoplasms and neoplasms
			INCB059872	Solid tumors and hematologic malignancies
		LSD1/HDAC6 dual inhibitor	JBI-802	Advanced and metastatic solid tumors
**SMRT/NCoR**	**TBL1**	Blocks TBL1/β-catenin interaction	Tegavivint	Solid tumors
**HDACs**	**HDACs 1, 2, and 3**	HDAC1, 2, 3, and 10 inhibitor	Chidamide	B-cell non-Hodgkin’s lymphoma
			combination therapies	Hodgkin’s lymphoma, non-Hodgkin’s lymphoma, peripheral T-cell lymphoma, cervical cancer, and diffuse large B-cell lymphoma
		Class I inhibitor	HBI-8000	Non-small cell lung cancer
		Class I and II inhibitor	Givinostat	Chronic myeloproliferative neoplasms
			OBP-801	Solid tumors
			AR-42	Vestibular schwannoma, meningioma, and acoustic neuroma
			Mocetinostat	Non-small cell lung cancer, diffuse large B-cell lymphoma, and follicular lymphoma
			Abexinostat	Follicular lymphoma, non-Hodgkin’s lymphoma, and diffuse large B-cell lymphoma
			Trichostatin A	Hematologic malignancies
			REC-2282	Neurofibromatosis
			Zabinostat	Advanced cancer
			Vorinostat	Advanced cancer, Ewing sarcoma, and Wilms tumor
			combination therapies	Colorectal malignant neoplasms, brain stem glioma, and cerebral astrocytoma
		Class I, II, and IV HDACi	Belinostat	Metastatic breast cancer and ovarian carcinoma
			Combination therapies	Urothelial carcinoma, adult T-cell leukemia, glioblastoma, and acute myeloid leukemia
			SAHA (vorinostat)	Melanoma and skin neoplasms
			Combination therapies	Malignant solid tumor, ovarian cancer, breast cancer, non-small cell lung cancer, neuroblastoma, diffuse intrinsic pontine glioma, anaplastic glioma, erythroid leukemia, hematologic malignancies, advanced cancers, and Cushing’s adenomas
			Romidepsin	Triple-negative breast cancer
			Combination therapies	Lymphoid malignancies and multiple myeloma
			Resminostat	Cutaneous T-cell lymphoma
		Pan-HDACi	Panobinostat	Nasopharyngeal carcinoma, lymphomas, and EBV+ solid tumors
			Combination therapies	Advanced solid tumors, multiple myeloma, melanoma, skin cancer, and chordomas
		Pan-HDAC inhibitor and DNA-damaging bendamustine	Tinostamustine	Small-cell lung cancer, soft tissue sarcoma, malignant melanoma, and triple-negative breast cancer
		Monocyte/macrophage targeted HDAC inhibitor	Tefinostat	Hepatocellular carcinoma
		HDAC activator	Entinostat	Neuroendocrine tumors, renal cell carcinoma, bladder cancer, breast cancer, breast adenocarcinoma, renal cell carcinoma, solid tumors, malignant solid neoplasms, metastatic and advanced cancers
**PRC2**	**EZH1/2**	EZH1/2 inhibitor	DS-3201b	Small cell lung cancer
			HH2853	Non-Hodgkin’s lymphoma
			PF-06821497	Small cell lung carcinoma, follicular lymphoma, and castration-resistant prostate cancer
		EZH2 inhibitor	SHR2554	Mature lymphoid neoplasms
			combination therapies	Solid tumor lymphoma
			Tazemetostat	Rhabdoid tumors, synovial sarcoma
			combination therapies	Metastatic prostate cancer, metastatic melanoma, cutaneous melanoma, hematologic malignancies, metastatic urothelial carcinoma, bladder cancer
			CPI-1205	B-cell lymphoma
			Combination therapies	Metastatic castration-resistant prostate cancer and advanced solid tumors
			CPI-0209	Advanced solid tumor, diffuse large B-cell lymphoma, and T-cell lymphoma
		EZH1/EZH2 dual inhibitor	Valemetostat tosylate	B-cell lymphoma
	**EED**	Blocks EED/EZH2 interaction	MAK-683	Diffuse large B-cell lymphoma

FDA, Food and Drug Administration; HDAC, histone deacetylase; HDACi, HDAC inhibitor; SAHA, suberoylanilide hydroxamic acid; PRC, polycomb repressive complex; EZH, enhancer of zeste homolog; CoREST, corepressor or REST; LSD, lysine-specific demethylase; SMRT, silencing mediator for retinoid or thyroid-hormone receptors; NCoR, nuclear receptor corepressor; TBL, transducing beta-like protein; EBV, Epstein–Barr virus; EED, embryotic ectoderm development.

^1^fda.gov

^2^clinicaltrials.gov.

Complex inhibition by blocking interactions/binding between complex members to disrupt complex formation is another strategy being explored to decrease Treg function. In the case of targeting components of the PRC2/1 complexes to increase anticancer immunity, inhibition of EZH2 has been effective against rare sarcomas and follicular lymphomas (**Table 2**). The expression of EZH2 has been associated with poor clinical outcomes in cancer patients, contributing to metastasis, metabolism, drug resistance, and angiogenesis (103), while conversely displaying tumor-suppressive functions (104). Cancer cells that express EZH2 have reduced CXCR9 expression (105) and decreased effector T-cell infiltration of tumors (106). Treatment of solid tumors with EZH2 inhibitors increases the recruitment and function of CD4+ and CD8+ effector T cells by induction of an inflammatory phenotype within tumor-infiltrating Tregs (107). Promising advances for this class of cancer therapeutics disrupt the complex formation of PRC2 rather than EZH2 enzymatic activity. Compounds A769662 (108) and MAK-683 (109, 110) accomplish this by blocking EZH2 interaction with PRC2 components SUZ12 and EED, respectively. MAK-683 is currently undergoing clinical trials for the treatment of diffuse large B-cell lymphoma (**Table 2**).

Other approaches in targeting Tregs for cancer interventions include activation of epigenetic regulatory complexes that function to repress Foxp3 and Treg function. For example, inositol phosphates activate the deacetylase activity of SMRT/NCoR, NuRD, and MiDAC (80, 84). Considering that NuRD and SMRT/NCoR have been found to induce Foxp3 expression in Tregs, the use of inositol phosphates could be particularly useful for the anticancer treatment or as part of a combination regimen (84).

Anticancer therapies targeting HDACs and EZH2 within epigenetic regulatory complexes have been individually effective at treating various cancer types and have received Food and Drug Administration (FDA) approval (**Table 2**). However, such therapeutics may potentially perform better in combination with drugs targeting separate cellular mechanisms. HDAC inhibitors used in combination with checkpoint inhibitors that target proteins such as PD-1/PDL1 or CTLA4 show therapeutic promise. Clinical trials for cervical cancer and cervical neoplasm with HDAC inhibitor toripalimab and PD-1 inhibitor chidamide. Such HDAC inhibitors can function to prime or sensitize cells for the checkpoint inhibitors to induce anticancer immunity. Another strategy for combination therapies involves the EZH2 inhibitor, SHR2554, currently in clinical trials as both an independent therapy and in combination with anti-PD-L1/TGFβ antibody, SHR1701. Further, in preclinical studies, EZH2 inhibition in Ewing sarcoma induces the expression of ganglioside G-D2, which is then targeted with gene-modified T cells, resulting in tumor regression (111). This is just one example of regulatory complex inhibition in combination with adoptive T-cell therapy, albeit a strategy gaining in popularity.

Anticancer therapeutics that target epigenetic regulation within Tregs have been effective clinically. While this has not been universally the case, our understanding, development, and employment of such therapeutics continue to progress, providing for more precise and effective therapeutic methods. Modulation of the epigenetic state of immune cells through targeting multiprotein regulatory complexes can involve new approaches. For example, in chimeric antigen receptor T (CAR-T) cell therapy, CAR-T cells could potentially be treated *ex vivo* with HDAC- or EZH2 complex-targeted inhibitors to stabilize their functional identity. A better understanding of the roles and mechanisms of large epigenetic regulatory complexes will advance the development of both conventional and innovative strategies for cancer immunotherapies.

While anticancer therapies continue to improve in efficacy and precision, obstacles persist. Collectively, these regulatory complexes are essential in establishing and maintaining cell lineage identity and therefore could be expected to be dysregulated in cancers. Indeed, this is usually the case; furthermore, mutations in the genes causing dysfunction often induce cancer development and contribute to cancer pathology (98). Dysregulation of the complexes and individual complex components, within cancer cells and tumor microenvironments, could result in altered effects of therapeutic inhibitors. In addition, the functions performed by these complexes are dynamic in that they vary in differing environments with and in response to specific regulatory landscapes. Functional variations are influenced by physical conformation, chemical modifications, and the availability and/or incorporation of cofactors to the complexes. The described plasticity of Foxp3 transcriptional regulation presents a barrier with respect to the prospect of Treg-specific therapeutic interventions (112), and designing and generating compounds to target large multiprotein complexes are inherently challenging (40). In addition, having various transcriptional functions in alternate cell types of these complexes, off-target effects provide additional obstacles to be considered. Indeed, there exists much room for improvement, and many obstacles persist, yet modulation of Treg function for therapeutic intervention against cancer is effective and continuously improving.

## Conclusion

As with other cell types, large multiprotein complexes are involved in the regulation of Foxp3+ Treg development, stability, and function. Except for MiDAC, about which little is known in terms of its immune functions, these complexes directly influence the transcription of Foxp3 gene. As coregulators, histone modification represses Foxp3 when facilitated by CoREST, Sin3a, and PRC2/1, while SMRT/NCoR and NuRD complexes enhance Foxp3 expression. The variation in function among the complexes deepens when individual subunits are taken into consideration, as shown by the opposing effects of HDAC1 and HDAC2 on Foxp3 expression in Tregs. Some of the complexes demonstrate multiple functions within Tregs. In addition to direct regulation of Foxp3 gene, CoREST, SMRT/NCoR, and PRC2 interact with the Foxp3 protein and contribute to the regulation of the Treg transcriptional program. These epigenetic regulatory complexes play important roles in the control of the transcriptional activity of Treg cells, which make them promising targets for the development of future anticancer therapeutic strategies.

## Author Contributions

LC drafted the review. LC and WH prepared the final version of the text. LC prepared the graphics. All authors listed have made a substantial, direct, and intellectual contribution to the work and approved it for publication.

## Funding

This work was supported in part by the National Institutes of Health (1R01CA253320).

## Conflict of Interest

The authors declare that the research was conducted in the absence of any commercial or financial relationships that could be construed as a potential conflict of interest.

## Publisher’s Note

All claims expressed in this article are solely those of the authors and do not necessarily represent those of their affiliated organizations, or those of the publisher, the editors and the reviewers. Any product that may be evaluated in this article, or claim that may be made by its manufacturer, is not guaranteed or endorsed by the publisher.
